# Life Cycle Assessment of Raw and Fe-Modified Biochars: Contributing to Circular Economy

**DOI:** 10.3390/ma16176059

**Published:** 2023-09-04

**Authors:** Carolina Gallego-Ramírez, Edwin Chica, Ainhoa Rubio-Clemente

**Affiliations:** 1 Grupo de Investigación Energía Alternativa (GEA), Facultad de Ingeniería, Universidad de Antioquia UdeA, Calle 70 No 52-21, Medellín 050010, Colombia; edwin.chica@udea.edu.co; 2 Escuela Ambiental, Facultad de Ingeniería, Universidad de Antioquia UdeA, Calle 70 No 52-21, Medellín 050010, Colombia

**Keywords:** circular economy, waste recycling, water treatment, carbonaceous material

## Abstract

Biochar is a carbonaceous material, which can be decorated with metals, that has been garnering attention to be used in the treatment of water due to its contribution to waste management and circular economy. This study presents the life cycle assessment (LCA) regarding the generation of *Pinus patula* raw biochar and its modification with iron (Fe-modified biochar). SimaPro 9.3.0.3 software was used to simulate the environmental impacts of both carbonaceous materials. The potential environmental effects obtained from the production of *Pinus patula* raw biochar were mainly ascribed to the source of energy utilized during this process. The potential impacts demonstrated that the generation of gases and polycyclic aromatic hydrocarbons are the main concern. In the case of Fe-modified biochar, the potential environmental effects differed only in the stage of the biomass modification with the metal. These effects are associated with the extraction of Fe and the generation of wastewater. These findings provide an insight into the environmental effects linked to the production of raw and Fe-modified biochar. However, further LCA research should be performed concerning other materials and compounds than can be generated during the biomass thermochemical conversion.

## 1. Introduction

Biochar is a carbon-rich byproduct derived from the production of energy; specifically, it comes from the thermal decomposition of biomass, which is often a waste [1]. In this regard, its utilization contributes to the management of waste and, subsequently, to sustainable development [2]. Any organic material can be used as biomass for biochar generation. Additionally, animal manure, sewage sludge, agro-industrial waste, and microalgae and wood residues are some of the biomass sources reported in the literature [3]. Hydrothermal carbonization, pyrolysis, torrefaction, and gasification are the processes through which biochar can be produced [4].

This carbonaceous material has been used in the treatment of soil and wastewater [5]. Especially for water treatment, biochar has been gaining attention as a novel adsorbent because of its low cost and different sources to be produced. Due to the uses of biochar in environmental supervision and multifaceted purposes, there has been a growing interest in its evaluation [6]. Biochar has different purposes, with some of these being the reduction in greenhouse gases, metal immobilization, nutrient exchange, the reduction and removal of organic and inorganic compounds in water, and carbon sequestration [7]. The use of modified biochar is also a topic of increasing interest among the scientific community, especially its modification to be used as a supporting material for iron (Fe) in heterogeneous Fenton processes, allowing the activation of hydrogen peroxide (H_2_O_2_), and in persulfate-based oxidation processes, enabling that of persulfate (S_2_O_8_^2−^) [8,9,10]. When biochar is used as a supporting material for Fe, the Fe-modified biochar can be used as a catalyst in the treatment of organic pollutants contained in water like emerging pollutants, dyes, and heavy metals [11,12,13,14,15]. Therefore, analyzing the potential environmental impacts associated could be of utmost interest, and for this purpose, life cycle assessment (LCA) is regarded as a crucial tool [16].

LCA is a method used to evaluate the ecological effects linked to the complete life cycle of a product, process, or activity. It presents a comprehensive approach that considers the sourcing of raw materials, production, utilization, and disposal or recycling of the product. LCA offers valuable insights into the environmental facets and potential consequences associated with a product or system throughout its life cycle [17,18].

Typically, it involves evaluating various impact categories such as greenhouse gas emissions, energy consumption, water usage, air pollution, and resource depletion. LCA offers a holistic view that empowers decision-makers to identify critical areas in the product or process life cycle and make informed choices to minimize environmental impacts [17]. It is relevant to note that for conducting a proper LCA, energy and material input data should be collected, as well as those ones related to soil, water, air, and waste generation. Other important factors related to each life cycle stage should also be considered [19].

These data are then analyzed using specific methodologies and tools to evaluate the potential environmental impacts and pinpoint opportunities for improvement. The life cycle of a product typically encompasses the following stages: (i) acquisition of raw materials; (ii) manufacturing; (iii) contribution and utilization; and (iv) end-of-life [20].

LCA finds widespread usage among industries, policymakers, and researchers to support sustainable decision-making, product design, process optimization, and environmental policy formulation [19,21]. It aids in identifying areas where enhancements can be made to reduce the overall environmental burden of products and processes, aiming for a more sustainable and efficient use of resources [22].

There are different software programs used to implement LCA such as SimaPro, GaBi, and Open LCA being some of those [23,24,25,26]. Among the mentioned software programs, SimaPro constantly updates its life cycle inventory data. It also includes different impact assessment methods and has the most updated Ecoinvent database library [27]. Additionally, SimaPro clearly displays the processes with the largest impact due to its powerful graphical interface [28]. Furthermore, for the valorization of waste into energy, which is the process where biochar comes from, the most used LCA software is SimaPro [29]. 

In the literature, there are several studies based on the LCA concerning the production of biochar. The studies present the differences that feedstock and biomass conversion temperatures can make in the potential environmental impacts associated with the production of the material [30]. Moreover, some studies are based on a comparison of the LCA results of activated carbon and biochar and other types of metal-based catalysts [30,31,32]. From the authors’ knowledge, this is one of the first studies assessing the LCA differences between raw biochar and Fe-modified biochar. 

In order to evaluate and optimize the environmental benefits and feasibility of biochar applications, the LCA of the biochar production must be carried out [25,33]. Additionally, considering the increasing interest in modifying biochar with metals to use it as a catalyst in the water treatment process, it is important to discern the added environmental impacts that the modification of biochar can exhibit. Considering the environmental impacts related to the production of metal-modified biochar and comparing them with those related to raw biochar, evaluating the feasibility of the use of biochar as a catalyst is a crucial aspect. Given the importance of contributing to sustainable development and circular economy by reducing environmental depletion, the decision of using a specific material should be performed after taking into consideration the environmental impacts concerning the material production, and not only its efficiency [26,34]. Studies of biochar LCA may differ on the functional unit and system boundaries, making the comparison of cross-studies difficult [26]. Hence, a study with a comparison of the production of two different biochars that use the same system boundary and functional units can make the comparison more accurate. 

Under this basis, the production of biochar for the water treatment, as well as the modified biochar with the process of metal impregnation, are analyzed from an LCA perspective using the SimaPro software to discern the difference in the potential environmental impacts between modified and raw biochar. For the raw biochar, the source of biomass is residual wood of the species *Pinus patula*, of which the biomass was decomposed via gasification in a top-lit updraft reactor. In the case of the modified biochar focusing on Chu et al. [11], the scenario is simulated as if the biomass is first treated with a solution Fe (II) chloride, dried, and then gasified following the same process used in the raw biochar production. Thus, the aim of this work is to compare the production of raw biochar with the production of Fe-modified biochar, considering that to produce the Fe-modified biochar, it requires an additional process and the use of a reagent that can influence the difference of both processes.

## 2. Methods and Materials

### 2.1. Software and Scope Definition

For the analysis of LCA, SimaPro 9.3.0.3. (PRé Sustainability, NL) software was used, which serves as a robust software solution for this purpose. Its extensive range of tools and functionalities empowers users to evaluate the environmental implications linked to the complete life cycle of goods, procedures, or undertakings [35]. By facilitating the gathering, examination, and comprehension of data, SimaPro facilitates a comprehensive appraisal of diverse impact classifications like energy utilization, emissions, waste production, and the depletion of resources. As stated above, the software aids in identifying crucial areas, streamlining procedures, and making well-informed choices toward fostering sustainability. Its intuitive interface and strong modeling capabilities render it an invaluable resource for industries, researchers, and policymakers committed to incorporating sustainability into their operational frameworks and decision-making mechanisms [36].

On the other hand, it is important to note that during the thermochemical conversion of biomass, such as *Pinus patula* wood pellets, solid waste is generated. This waste is also called as biochar and can be used for several purposes, like a pollution treatment material. This carbonaceous solid can be also decorated with metals including Fe, allowing for increasing the efficiency of the raw biochar. Considering the increasing attention carbonaceous materials are garnering within the scientific community for treating polluted water and wastewater, such as that from the textile industry, raw and Fe-modified biochars are considered as adsorbing materials in the LCA, contributing to circular economy. The production of both biochars and their characteristics are covered below. 

In this regard, the goal and scope of this study consist of determining and comparing the potential environmental impacts associated with the production of *Pinus patula* wood pellets of raw and Fe-modified biochars. 

**Figure 1:**
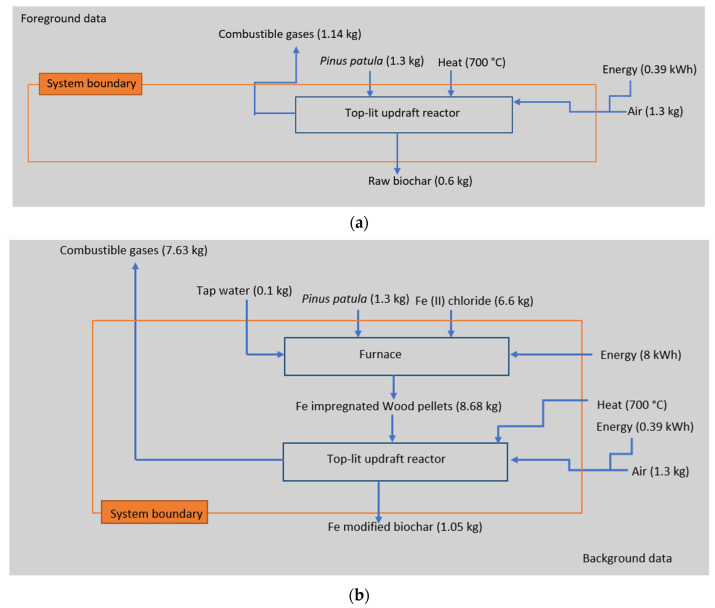
System representing the production of (**a**) raw biochar and (**b**) Fe-modified biochar. Conditions: P = 640 mmHg, T = 25 °C.

Figure 1a,b shows the system boundaries, inputs, outputs, and the two methods of biochar production that are compared. As illustrated, the system boundary does not include the extraction of raw materials (*Pinus patula* wood pellets). The decision not to include it was made because the wood used to produce biochar is wood waste. Additionally, the extraction of the raw material was left behind the system boundaries because biochar was already made, meaning that the type of biomass and the operational conditions in the biochar production cannot be changed; this is the reason why the air, energy, and heat inputs to the production of biochar were left behind too. Moreover, the output of combustible gases was not included in the system boundaries because even though the production of combustible gases is the main product when biomass is thermochemically decomposed, in this case, the product of interest is biochar. The product manufacturing (biochar generation) was included in the system boundary. Concerning the modification of biomass with Fe, again, the extraction of the raw material was not included for the wood pellets for the same reason mentioned above as ascribed, i.e., since it was a wood waste, it was not considered to be included, and for the tap water and Fe(II) chloride, since the information about these inputs is based on the work carried out by Chu et al. [11], that information cannot be changed. 

The allocation used in the process LCA is a mass allocation because mass helps to discern the biomass amount that is converted into biochar and combustible gases. The environmental impacts were calculated based on the process used in the production of both biochars. Since the modification of biochar has been gaining attention recently [37], it is important to compare what added impacts has to the process of biochar-modified production when compared to the raw biochar production. 

Based on the functional unit, which is 1 kg of biochar, the impact categories assessed were: global warming potential, human toxicity potential, ecotoxicity potential, and ozone depletion potential. 

### 2.2. Inventory Analysis

To produce the biochar, residual wood of *Pinus patula* in the form of pellets was used. The gasification process was carried out in a top-lit updraft reactor operated at atmospheric pressure. As the gasification agent, air from a reciprocating compressor was utilized [38,39].

For the Fe-modified biochar, the biomass was previously modified. The modification was assumed to consist of a metal impregnation using Fe(II) [11]. This type of modification has been used to combine biochar with advanced oxidation processes in the treatment of aquatic matrices [40]. 

The mass of the biomass that enters the system was 1.3 kg, and it was converted into 0.16 kg of biochar and 1.14 kg of combustible gases under a temperature of 700 °C. The gases obtained during the biomass gasification were composed of methane (CH_4_), carbon monoxide (CO), hydrogen (H_2_), hydrocarbons, carbon dioxide (CO_2_), and nitrogen (N_2_) [38]. It is highlighted that the software SimaPro 9.3.0.3. does not have the option to enter heat in terms of degrees. When biomass was used as an energy source and the gasification process was performed at a low-scale adaptation, the gasification process produced less than 20 kW [39]. Due to this, the heat entering the system was assumed as 15 kW, which was also assumed to be produced via a furnace operating with wood biomass as the energy source. Regarding the air, the airflow was 146 L/min. To obtain the mass of air that enters the system, assuming air as an ideal gas, with the average atmospheric pressure (*P*) and temperature (*T*) in Medellín (640 mmHg and 25 °C, respectively), the air density can be calculated by using Equation (1).

(1) $$\rho =\frac{P}{R\times T}$$

where ρ is the air density, *P* is the atmospheric pressure, *R* is the gas constant, and *T* is the air temperature.

Then, considering 544 s as the residence time, the density, and the volumetric flow of air, the air mass was calculated as 1.32 kg of air. The energy required by the reciprocating compressor was assumed to be 2.60 kW, where this energy was entered as the market for electricity, and the low voltage in Colombia as the product of the energy required by the compressor and the residence time. Hence, the system energy input was 0.39 kWh. 

For the scenario of the Fe-modified biochar, to impregnate the biomass with Fe, 6.6 kg of Fe(II) chloride, 0.1 kg of tap water, and a furnace that requires 4 kW for 2 h were assumed to be used to simulate the modification of the biomass according to Chu et al. [11]. Since the biomass absorbs the tap water and Fe(II) chloride, in this scenario, the biomass input in the top-lit updraft reactor was assumed to be 8.68 kg. The energy, the air mass, and the heat remained the same as the ones used in raw biochar production. The biochar yielded in the gasification of *Pinus patula* wood pellets was 12.12 wt% [38]. Therefore, the mass of the Fe-modified biochar was defined as 1.05 kg of the initial Fe-modified biomass, and the remaining value was attributed to the combustible gases. 

The production and physicochemical properties of *Pinus patula* wood pellets’ raw biochar are described in detail in the work conducted by Gutiérrez et al. [38,41]. In the case of the Fe-modified biochar, the generation process is explained by Chu et al. [11]. According to this study, the percentage of iron in the modified biochar is expected to be approximately 48.8 wt.%.

Furthermore, in the analysis of the environmental impacts associated, the data were collected from previous studies and information found in the literature about the production of raw and Fe-modified biochars. Based on the impact assessment categories that were chosen to be evaluated in this study (i.e., global warming potential, human toxicity potential, ecotoxicity potential, and ozone depletion potential), the ReCiPe 2016 (PRé Sustainability, NL) and the USEtox (UNEP/SETAC, KE) impact assessment methods were implemented to assess the potential environmental impacts associated with the production of both adsorbing materials. 

## 3. Results and Discussion

### 3.1. Impact Assessment

For the impact assessment and the categories analyzed in the study, Eco-indicator 99 (PRé Sustainability, NL) is described to be useful to evaluate those impact categories [42]. Nevertheless, in SimaPro 9.3.0.3., the above-mentioned method does not have the option to be chosen as a global method. Moreover, it was found that Eco-indicator 99 only considers European processes, which can be different from other processes applied worldwide. In addition, the characteristics used to calculate the environmental impacts are based on the environmental conditions in Europe [43]. Therefore, the ReCiPe 2016 method was selected to discern the environmental impacts. This decision was ascribed to the environmental impact categories of global warming potential, human toxicity potential, ecotoxicity potential, and ozone depletion potential included within. Furthermore, ReCiPe 2016 allows the selection of a global method [44]. This method was chosen in the hierarchist (H) version since it includes a long perspective of the potential impacts of the process [45].

On the other hand, the USEtox method was used to calculate the toxicity (human cancer and non-cancer) and ecotoxicity impact categories. The decision to use USEtox underlies the potential detrimental consequences on human health and the ecosystem from the realization of chemicals into the environment [46].

**Figure 2:**
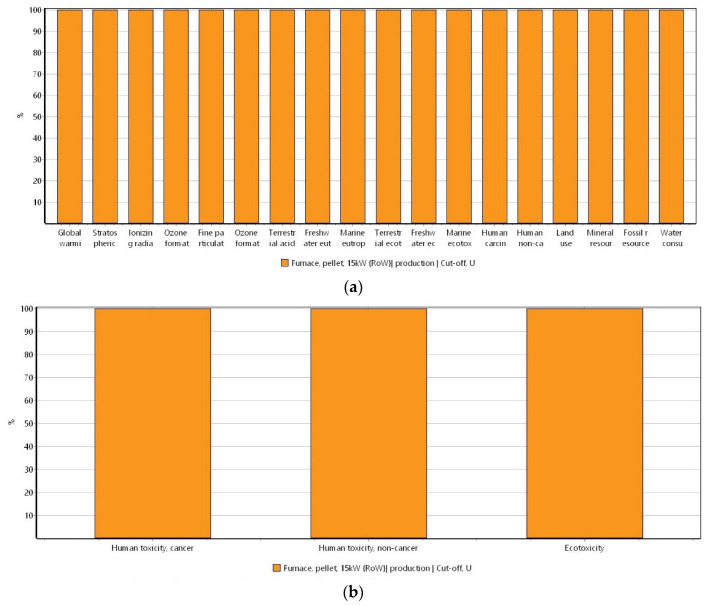
Potential environmental impacts ascribed to the production of biochar by using (**a**) ReCiPe 2016 method (ReCiPe 2016 Midpoint (H) V1.06/World (2010) H/Characterization) and (**b**) USEtox method (USEtox V 1.01/Characterization). System unit: 1 kg of biochar.

Figure 2a shows the results of the potential environmental impacts derived from the raw biochar generation by using the ReCiPe 2016 method. In turn, in Figure 2b, the results derived from the USEtox method are depicted. 

**Figure 3:**
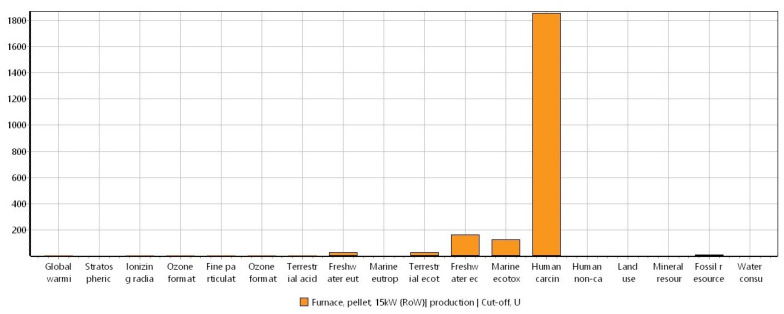
Normalization of the potential environmental impacts ascribed to the biochar production by using the ReCiPe 2016 method (ReCiPe 2016 Midpoint (H) V1.06/World (2010) H/Normalization). System unit: 1 kg of biochar.

In Figure 3, the normalization of the environmental impacts in the production of biochar obtained from the ReCiPe 2016 method is represented. 

**Figure 4:**
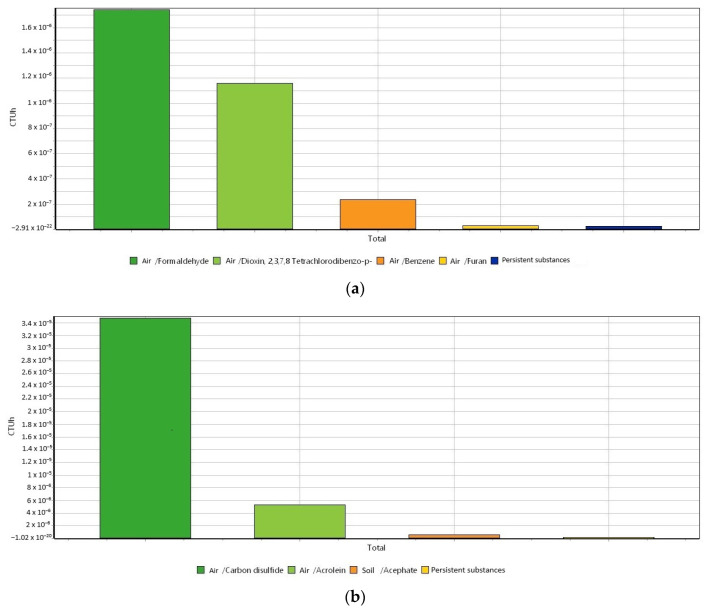

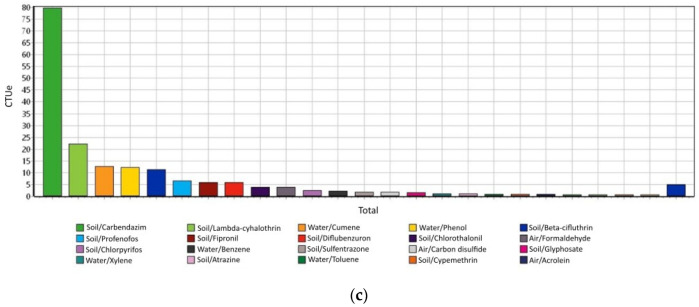
USEtox method (USEtox/Characterization) for the three impact categories ascribed to the production of biomass. (**a**) Human toxicity cancer, (**b**) human toxicity non-cancer, and (**c**) ecotoxicity. System unit: 1 kg of biochar.

Since the USEtox method can display the impacts as different harmful components, Figure 4a–c shows different plots for the three environmental categories of human toxicity cancer, human toxicity non-cancer, and ecotoxicity, respectively, to produce raw biochar.

**Figure 5:**
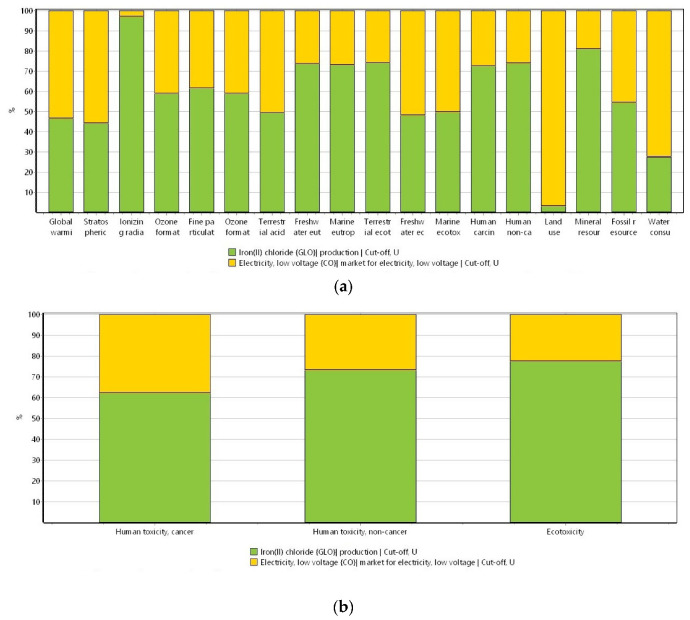
Potential environmental impacts derived from the modification of biomass by using (**a**) ReCiPe 2016 method (ReCiPe 2016 Midpoint (H) V1.06/World (2010) H/Characterization) and (**b**) USEtox method (USEtox V 1.01/Characterization). System unit: 1 kg of Fe-modified (**a**) biomass and (**b**) biochar.

**Figure 6:**
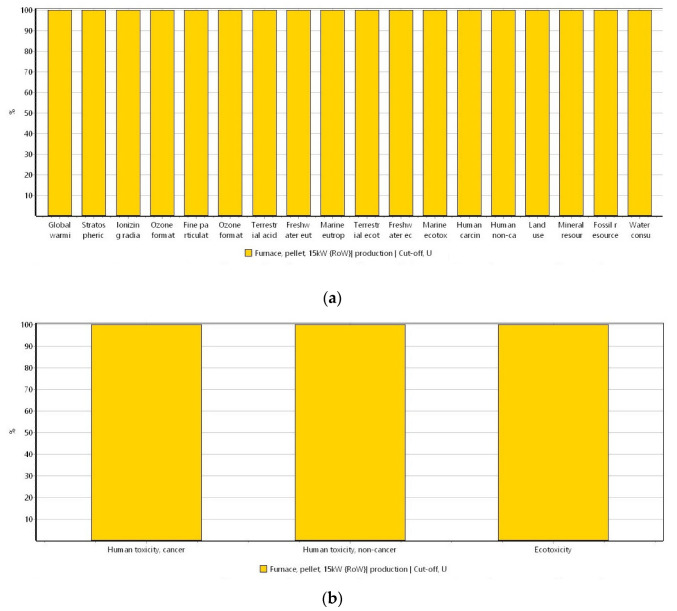
Potential environmental impacts ascribed to the production of Fe-modified biochar by using (**a**) ReCiPe 2016 method (ReCiPe 2016 Midpoint (H) V1.06/World (2010) H/Characterization) and (**b**) USEtox method (USEtox V 1.01/Characterization). System unit: 1 kg of Fe-modified biochar.

Concerning the Fe-modified biochar, the potential impacts derived from the biomass modification were assessed first. Subsequently, the impacts of Fe-modified biochar production were evaluated. Figure 5 shows the potential environmental impacts assumed to be produced from biomass modification. Figure 6 refers to the impacts derived from the Fe-modified biochar.

**Figure 7:**
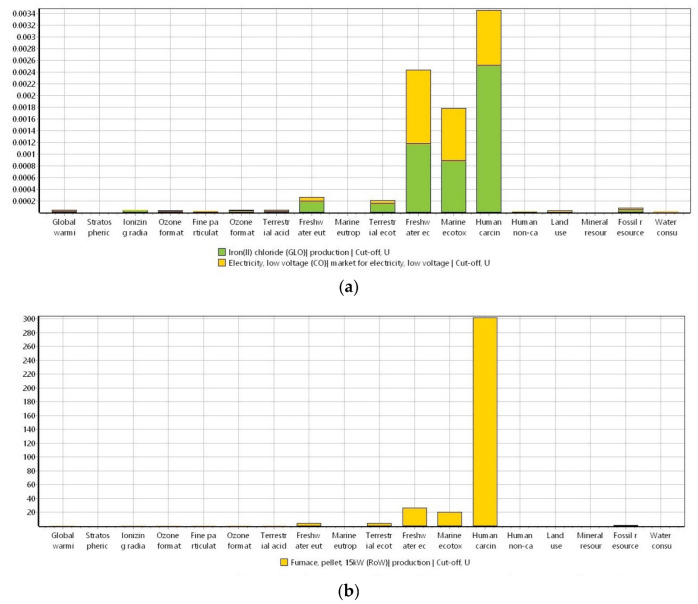
Normalization of the ReCiPe 2016 method (ReCiPe 2016 Midpoint (H) V1.06/World (2010) H/Normalization) for (**a**) the biomass modification and (**b**) the production of the Fe-modified biochar. System unit: 1 kg of Fe-modified (**a**) biomass and (**b**) biochar.

**Figure 8:**
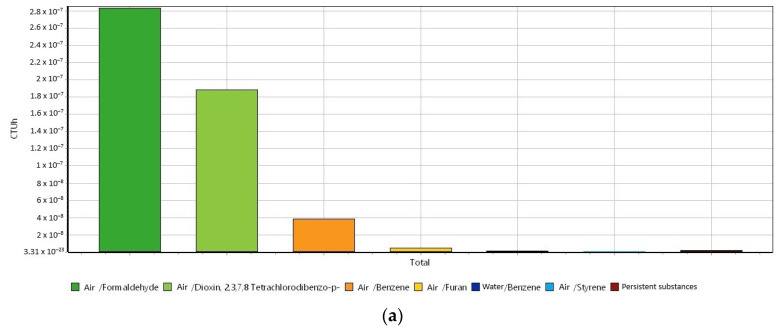

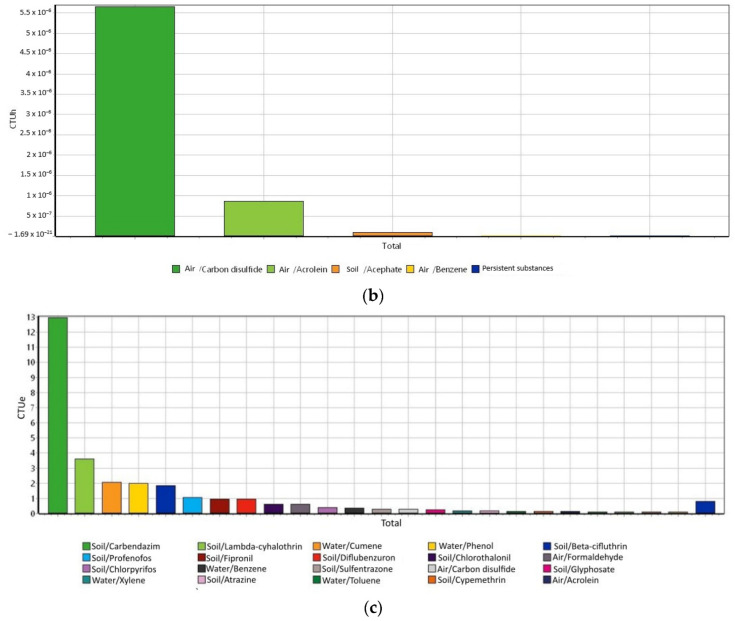
USEtox method for the three impact categories derived from the production of Fe-modified biochar (USEtox/Characterization). (**a**) Human toxicity cancer, (**b**) human toxicity non-cancer, and (**c**) ecotoxicity. System unit: 1 kg of Fe-modified biochar.

Regarding the biochar production, the impacts derived from the biomass modification and the generation of the Fe-modified biochar plots for the normalization of the ReCiPe 2016 method were obtained (Figure 7). Concerning the environmental impacts ascribed to the production of Fe-modified biochar, they are plotted for each one of the categories considered in the USEtox method (Figure 8). 

### 3.2. Impact Interpretation

To produce the raw biochar and the impact categories of the ReCiPe 2016 and the USEtox methods (Figure 2a and Figure 2b, respectively), it can be seen that all the environmental impacts are attributed to the energy source utilized during the generation of biochar. This can be explained because when wood is used to produce energy, carbon dioxide (CO_2_) and carbon monoxide (CO) emissions can be produced. Additionally, the gasification process can lead to the formation of polycyclic aromatic hydrocarbon (PAH) compounds that are widely known for the impacts that can generate in both the environment and the living organisms. Furthermore, the production of energy from the biomass increases carbon emissions [47,48,49]. Likewise, since the biomass used to generate biochar is a residual wood, other than making a negative impact, this is involved in positive impacts because a generated waste during a specific process is used in another process. On the other hand, the energy required by the reciprocating air compressor is for a short period of time; therefore, the impacts that generally come with the use of energy are almost imperceptible. 

When the ReCiPe 2016 method was displayed as a normalization of the impact categories (Figure 3), the categories of human carcinogenic, freshwater, and marine ecotoxicity stood out among the others. This can be explained due to the carcinogenic, mutagenic, and teratogenic characteristics derived from the exposure to PAH in the environment [50,51]. In addition, these compounds exhibit high ecotoxicity [52], meaning that the impacts generated in the categories of freshwater and ocean ecotoxicity can also be attributed to the generation of these compounds during biomass gasification [53]. 

For the human toxicity cancer category withdrawn from the USEtox method (Figure 4a), the emission of formaldehyde is the main hotspot in the category. This can be explained because biomass gasification is considered a formaldehyde source [54]. The toxicity of formaldehyde is attributed to its carcinogenic, mutagenic, and reprotoxic ability [55]. The second hotspot in the human toxicity cancer category is the emission of the dioxin, as well as PAH, which in turn can be generated in biomass gasification. Dioxins are also known as carcinogenic compounds. For the category of human toxicity non-cancer (Figure 4b), the main hotspot is the emissions of carbon disulfide. This can be attributed to the carbon emissions that take place in the production of energy via biomass. According to the United States Environment Protection Agency (EPA), carbon disulfide can cause neurologic effects, headache, dizziness, fatigue, and other symptoms when humans are exposed to the compound [56,57]. For the ecotoxicity in the production of biochar (Figure 4c), the discharge of carbendazim to soil is the main hotspot. Carbendazim is a pesticide, and a vast number of research linked to biochar and carbendazim are about the adsorption of carbendazim into biochar [58,59,60,61]. From the authors’ knowledge, the production of biochar has not been found to be linked to carbendazim production or pollution. The biochar production ecotoxicity can be related to the production of PAH that turns out to have ecotoxic effects in the environment and can bioaccumulate and biomagnificate within the fat tissues of living organisms. 

For the modification of biomass with Fe(II) chloride, in Figure 5a, it is seen that for the ReCiPe 2016 method, environmental impacts are mainly attributed to the Fe(II) chloride. This can be explained because of the environmental impacts of the extraction of iron since this industrial activity produces a large amount of wastewater and gas emissions [62]. For the USEtox method (Figure 5b), again, the use of Fe(II) chloride exhibits the highest influence. As shown in Figure 6, the potential environmental impacts do not differ from the raw biochar production to the Fe-modified biochar generation; hence, the addition and the difference in the environmental impacts is seen in the biomass modification but not in the production of the Fe-modified biochar. In Figure 7a, the categories of human toxicity, freshwater, and ocean ecotoxicity for biomass modification represent the main impacts during this process, which are mostly attributed to the use of Fe. As it was described previously, this can be derived from the liquid and gaseous emissions generated during the extraction of iron that can lead to air and water pollution bodies, subsequently affecting acoustic ecosystems, including fauna, flora, and humans [63]. 

As shown in Figure 7b and Figure 8a–c, the environmental impacts that the production of Fe-modified biochar can incur are the same as the ones derived from the production of the raw biochar. Thus, the additional impacts generated in the production of modified biochar are mostly common to appear during the modification phase of the biomass. This can be attributed to the use of different metals (including Fe) that can cause additional pollution of the environment.

### 3.3. Future Perspectives

It is important to note that the application of biochar in the treatment of water has some challenges, such as (i) assessing the impacts and role of biochar in the treatment of water and its further disposal; (ii) considering the type of feedstock, temperature, and the modification method is imperative before implementing a large-scale application of biochar, since the environmental impacts depend on these factors; (iii) taking into account the positive or negative climate effects associated with the production of biochar is crucial to evaluate the feasibility of the production and subsequent use of biochar [34]. 

To overcome these challenges, an LCA referring to biochar use in the treatment of wastewater and water and the possible disposal method should be carried out. This will help infer the impacts ascribed to the utilization of biochar as an adsorbent for water treatment. Additionally, evaluating different disposal methods for biochar should represent an insight into the existing gap regarding the disposal of the material. On the other hand, the LCA of various biochar production processes may allow us to choose the best biochar production method and, subsequently, the starting point for the industrial production and use of biochar. Furthermore, considering the effects of climate change generated by biochar could refer biochar to its industrial application, LCA is one of the tools for analyzing the impacts on climate change associated with the production of biochar. 

## 4. Conclusions

The potential environmental effects related to the production of both raw and Fe-modified biochars via gasification are mainly attributed to the energy source used in the production of biochar, and, subsequently, to the gasification process itself. Since the gasification of wood is known to produce gas emissions like CO_2_, CO, and PAHs, the impact categories for this stage of the production of biochar stand out in comparison to the other stages.

When analyzing the LCA of a process, it is often found that the use of electricity is derived in high environmental impacts due to the depletion of resources. Nevertheless, in this study, it was found that the use of electricity does not represent potential environmental impacts, since it was used for a short period of time. In this regard, the potential impacts were exceeded via the gasification of wood. 

Additionally, the environmental consequences associated with the manufacturing of Fe-modified biochar are comparable to those arising from the production of unmodified biochar. Consequently, the supplementary impacts generated during the production of modified biochar predominantly arise in the biomass modification stage. This can be attributed to the utilization of diverse metals, including Fe, which can result in additional environmental pollution. This environmental pollution would be related to the processes carried out to extract diverse metals like Fe, which are well-known methods to cause high environmental depletion mainly in the soil matrix. Moreover, in the case of this study, since the biomass modification is performed in an aqueous medium, there is a risk of producing wastewater containing metals. Therefore, to reduce the impacts of Fe-modified biochar, a treatment of the residual water containing Fe must be taken into consideration so that a modified material can be produced with non-added impacts compared to the raw material or at least with a reduced number of side impacts. 

Furthermore, the production of raw and Fe-modified biochars can represent potential environmental effects mainly attributed to the generation of toxic substances during the production of biochar. Hence, further research should be performed to study and explain the contribution of biochar to environmental depletion and the toxicity related to its generation and use. 

## Data Availability

Data are contained within the dcoument.
